# Exploration of the psychological impact and adaptation to cardiac events in South Asians in the UK: a qualitative study

**DOI:** 10.1136/bmjopen-2015-010195

**Published:** 2016-07-08

**Authors:** Mimi Bhattacharyya, Fiona Stevenson, Kate Walters

**Affiliations:** Research Department of Primary Care and Population Health, University College London, London, UK

**Keywords:** REHABILITATION MEDICINE, QUALITATIVE RESEARCH, PRIMARY CARE

## Abstract

**Objective:**

There is little research on how different ethnic groups adapt after an acute cardiac event. This qualitative study explores between-ethnicity and within-ethnicity variation in adaptation, and the psychological impact of an acute cardiac event among UK South Asian and white British people.

**Setting:**

We purposively sampled people by ethnic group from general practices in London who had a new myocardial infarction, angina or acute arrhythmia in the preceding 18 months.

**Participants:**

We conducted 28 semistructured interviews for exploring the psychological symptoms, experiences and adaptations following a cardiac event among South Asians (Indian and Bangladeshi) in comparison to white British people. Data were analysed using a thematic ‘framework’ approach.

**Results:**

Findings showed heterogeneity in experiences of the cardiac event and its subsequent psychological and physical impact. Adaptation to the event related predominantly to life circumstances, personal attitudes and employment status. Anxiety and low mood symptoms were common sequelae, especially in the Bangladeshi group. Indian men tended to normalise symptoms and the cardiac event, and reported less negative mood symptoms than other groups. Fear of physical exertion, particularly heavy lifting, persisted across the groups. Some people across all ethnic groups indicated the need for more psychological therapy postcardiac event. Socioeconomic circumstances, age and prior work status appeared to be more important in relation to adaptation after a cardiac event than ethnic status.

**Conclusions:**

Heterogeneity in views and experiences related to the socioeconomic background, age and work status of the participants along with some cultural influences. Rehabilitation programmes should be flexibly tailored for individuals in particular and where relevant, specific support should be provided for returning to work.

**Strengths and limitations of this study**

- We sampled to ensure a broad range of views and experiences from patients within a wide range of ages, employment situation and from a variety of socioeconomic backgrounds and education.
- We used trained interviewers who spoke Sylheti to enable participants to talk at length in their own language.
- The main researcher could understand Bengali and the related dialect Sylheti, and could verify the accuracy of the interpreter's work and prompt probing of responses.
- A diverse range of views were reported, including both positive and negative examples of adaptation.
- The South Asian community is culturally heterogeneous; therefore, caution is needed with data interpretation on cultural issues.

## Introduction

South Asians (people from India, Pakistan and Bangladesh) have higher prevalence and incidence of coronary heart disease (CHD).^1^ In comparison with white British groups, South Asians exhibit both a biological profile of increased cardiovascular risk^2^ and an adverse psychosocial profile of increased cardiovascular risk.^3^ ^4^ A diagnosis of CHD can have psychological, physical and social consequences^5^ which may require considerable adjustment from the individual in various life domains.

Adaptation is most commonly defined as absence of psychological distress, and involves the related components of preserving functional status, quality of life and absence of psychological symptoms as well as retaining a purpose in life and positive outlook.^6^ Some people who face the stress of a serious illness adjust well, whereas others may show significant psychological distress.^7^ Psychological distress, including anxiety and depression, can result in impaired social functioning and quality of life, impeding both psychological and physical recovery. Depression is relatively common in patients with heart disease, and associated with an increased risk of mortality and morbidity.^8^ ^9^

Studies show there is considerable heterogeneity in adaptation following heart disease^10^ ^11^ and to date, there are few studies exploring the influence of ethnicity on adaptation and psychological well-being. A cross-sectional population study exploring psychosocial risk factors and ethnicity concluded that UK South Asian men and women report significantly higher psychosocial adversity compared with the white UK population.^12^ A review article^13^ emphasised the lack of concordance between incidence of actual CHD and prognosis in South Asians. There is some research to support adverse prognosis in the immediate aftermath of a heart attack^14^ ^15^ in comparison to Caucasian patients; however, a recent retrospective database analysis study^16^ examining outcomes after cardiac angiography concluded that outcomes for South Asians were no worse than those for Caucasians.

A quantitative study from Australia suggested that there are higher levels of psychiatric symptom presentation in South Asian groups, with the suggestion that depression may be underdetected and this may be contributing to adverse outcomes in these groups.^17^ In a qualitative study interviewing Yoruba, Bangladeshi and white British people, it was suggested that cultural models of depression are diverse and differ between ethnic groups.^18^

In relation to the use of services, women, older people and ethnic minorities may be less likely to attend a rehabilitation programme and if they do so, less likely to complete it.^19^

While there are several quantitative studies measuring the influence of ethnicity on outcomes after heart disease, there are few qualitative studies exploring the reasons why differences may occur. There is little research exploring adaptations across multiple life domains after heart disease between different ethnic groups. We explored illness perceptions, beliefs, health behaviours, psychological symptoms, experiences and adaptations after heart disease among South Asian Indian and Bangladeshi groups and white British people with the aim of identifying interethnic differences in the psychological impact and adaptations made after an acute cardiac event.

### Participants and methods

#### Design

Qualitative semistructured interviews.

#### Setting

Nineteen general practices were purposively selected from six inner and outer boroughs in Central, North and East London to reflect the ethnic diversity found in these areas and represent white British, Indian and Bangladeshi populations.

#### Study population and sample

Purposive sampling was used to select people according to ethnic group from those who have had an acute admission and undergone a cardiac intervention for an acute coronary syndrome or ventricular arrhythmia (angioplasty or stent or device insertion) or thrombolysis for an acute myocardial infarction or investigated and treated for angina within the previous 18 months. We excluded those who were too unwell to complete an interview. We focused on three groups in total: two South Asian groups (Indian and Bangladeshi) and white British. The following characteristics were monitored to ensure maximum diversity of the sample: age (working and non-working population aged 40 years and over), gender and socioeconomic class. Sampling continued aiming for saturation on main themes (no new themes emerging).

#### Data collection

Potential participants were identified from 19 participating general practitioner (GP) practices by the practice staff and sent a letter from their practice, signed by their GP, asking if they would be willing to consider taking part in an interview. Non-responders were sent one further reminder letter.

A topic guide for the interviews was developed by the research team based on findings from relevant literature. The topic guide was piloted with two South Asian participants who were not included in the study and amended accordingly. Written informed consent had been obtained from the participants following which semistructured interviews of selected patients were conducted by MB. Non-English-speaking patients were interviewed by interpreters trained in conducting qualitative interviews and recruited from a specialist company. These interviews were then transcribed into English. The main researcher (MB) speaks Bengali and can understand the related dialect of Sylheti, used by some Bangladeshis. She was present at all the interviews and could follow the dialogue. This acted as a quality check to ensure comparability across all the interviews. Interviews broadly explored how participants conceptualised, understood and expressed the nature of their symptoms during and after the initial cardiac event, and their adaptations emotionally, physically and functionally, that is, the return to work, lifestyle changes and new concerns. We explored this in the context of cultural and personal factors contributing to any psychological distress experienced, and any professional services or alternative and informal help-seeking strategies or internal resources used by participants. The interviews were held at a time and place convenient to the patient, and lasted for ∼35–45 min. Interviews were audio tape recorded, and transcribed and the field notes were completed. Participants were offered a £20.00 voucher for their participation.

#### Data analysis

Analysis was undertaken using the ‘framework’ analysis^20^ identifying key themes and their meanings. This is a widely used approach, particularly used for healthcare evaluations. Verbatim transcripts (including those interviews which were interpreted and then transcribed into English) were independently reviewed by members of the research team. The team contained some ethnic diversity and was multidisciplinary. A thematic framework was developed identifying key issues, concepts and themes. The framework was independently applied by the researchers to the transcripts and refined by consensus. The data were charted using Excel to build a picture of the whole data set. The framework approach allowed for a ‘within case’ (rows) and ‘across case’ (columns) analysis. Participants own language was used when shortening comments and referencing extracts back to the original transcripts. In the final interpretation stage, the entire data set was mapped and interpreted by the study team as a whole. Interpretation and analysis at all times remained grounded in the data collected. Framework analysis was selected as a particular strength of this method is in facilitating examination of the data across and between cases for patterns and connections, allowing for consideration of interethnic and intraethnic differences in the psychological impact and adaptations made after an acute cardiac event.

## Results

### Participant characteristics

A total of 201 people were found to be potentially eligible, and 29 responded of whom 28 were interviewed.

Sample characteristics consisted of 28 participants: 10 white British, 10 Indians and 8 Bangladeshis aged 44–88 years (mean age 66.6 years; SD 12.2) and predominantly male; 23 males compared with 5 females (2 of whom were Bangladeshi, 3 were white British). Mean age was 72.3 years for the Caucasian group, 68 years for the Indian group and 58 years for the Bangladeshi group. Thirteen out of the 28 were retired (those retired from a professional background included 4 from the Indian group, 1 from the Caucasian group and none from the Bangladeshi group); the remainder described a variety of occupations, for example, taxi drivers, managers, small business owners and homemakers. Twenty out of 28 lived in private housing. All eight Bangladeshi participants resided in council-owned housing and were all recruited from a socioeconomically deprived area in East London. Twenty-three participants out of 28 were married, 1 was a divorcee and 4 were single. All 10 Indian participants were Hindu and all 8 Bangladeshi participants were Muslim.

This is illustrated in table 1.

**Table 1:** Summary of participant demographic characteristics

ID	Age	Gender	Group	Occupation (OPCS classification)	Housing
1	74	Female	Caucasian	Retired (skilled non-manual)	Private flat
2	80	Male	Caucasian	Retired	Private flat
3	85	Male	Caucasian	Retired (skilled non-manual)	Private flat
4	81	Male	Caucasian	Retired (skilled non-manual)	Private flat
5	88	Female	Caucasian	Retired (professional)	Private house
6	55	Male	Caucasian	Partly skilled	Private house
7	59	Male	Caucasian	Skilled non-manual	Private flat
8	63	Male	Caucasian	Professional	Private house
9	83	Female	Caucasian	Unskilled	Private house
10	55	Male	Caucasian	Skilled non-manual	Private house
11	76	Male	Indian	Retired professional	Private house
12	55	Male	Indian	Skilled non-manual	Private house
13	62	Male	Indian	Skilled non-manual	Private house
14	67	Male	Indian	Retired (skilled non-manual)	Private house
15	82	Male	Indian	Retired (skilled manual)	Private house
16	71	Male	Indian	Retired (professional)	Private house
17	79	Male	Indian	Retired (skilled non-manual)	Private house
18	61	Male	Indian	Skilled non-manual	Private house
19	75	Male	Indian	Retired (professional)	Private house
20	52	Male	Indian	Skilled non-manual	Private house
21	73	Male	Bangladeshi	Retired (skilled manual)	Council house
22	50	Male	Bangladeshi	Unemployed (skilled manual)	Council flat
23	63	Female	Bangladeshi	Housewife	Council house
24	61	Female	Bangladeshi	Housewife	Council house
25	53	Male	Bangladeshi	Partly skilled, on long-term sick leave	Council house
26	57	Male	Bangladeshi	Partly skilled, on long-term sick leave	Council house
27	61	Male	Bangladeshi	Skilled non-manual	Council house
28	44	Male	Bangladeshi	Skilled manual	Council flat

Experiences of the acute event and how it presented varied considerably in terms of symptoms and severity, as well as perception of the event. Little relationship was found between the reported disease severity and the psychological and physical sequelae. There were no ethnic differences in terms of reported severity of symptoms.

Exploring how patients adapted to the event involved exploring several themes in relation to adaptation.

The results are grouped into five themes that consider reports of adaptation across (1) the psychological and (2) physical domains, (3) attitudes to the future, (4) support for adaptation and (5) described adaptations.

### Psychological impact of CHD

#### Emotional sequelae—low mood, anxiety and fear

There was heterogeneity in views across participants, including initial shock at the diagnosis, followed by reported low mood, anxiety and resentment at feeling limited in physical capabilities in the aftermath of the cardiac event. Immediate feelings after discharge included continued shock and anger, and feelings of ‘life being unfair’ and feeling like an ‘invalid’. Concerns were also expressed about mortality after what was perceived to be a life defining moment and reports of feeling emotionally labile.

Shock. Inconvenient. Most I thought was…this is, I don't need this! You know, this is just really inconvenient. And also I'd been careful with my diet. I don't smoke. I don't drink. I've been taking lots of herbal. I thought this just isn't fair. I haven't, what have I done? I'm gone, that's it. I was extremely, tearful, well people are tearful after a heart attack. (Male_1_, white British, aged 50s)

People also reported experiencing conflicting emotions.

on the way home in the car I was just I think very upset but glad to be alive at the same time, if that makes sense…and I said, ‘Oh yeah, I just can't believe this has happened,’ and everything else. (Male_2_, white British, aged 50s)

Seven out of the eight Bangladeshi participants' accounts expressed psychological symptoms such as feelings of being unable to cope or sleep due to worry and fear of a future cardiac event, having ‘a weak heart’ and not being mentally strong.

…he was so tense that you know he's still in the night he can't sleep, you know, in the middle of the night he wake up and then sit and thinking about that any time he could have the heart attack again, it will come back again. (Male_3_, Bangladeshi, aged 50s, translated)

Age was an important factor for variations in expressed negative emotional sequelae. Fewer negative psychological sequelae were reported by people who were retired, and this was true for all the ethnic groups. This may be due to different perspectives of the future.

if I did go that way it's a wonderful way to go, because you're not suffering. (Female_4_, white British, aged 80s)

#### Changing role perception of self and how others perceive them

There was a self-perception among some participants that they were weaker, both mentally and physically. This resulted in them adapting by avoiding conflict to reduce stress levels.

If anybody wants to hit me for nothing, or to push me, I would say, ‘I'm right,’ but I would say, ‘I am sorry.’ Because mentally I thought I am not strong enough, I've got a weak heart. (Male_5_, Bangladeshi, aged 40s)

Work was reported to be an important area relating to self-esteem in all three groups regardless of ethnicity or occupation. A particular difficulty was the challenge to the role of ‘provider’ for the family.

I don't want to be here as an invalid. I would rather be gone. And I mean that from the heart. For my family, they can't understand that. I want to be psychologically, mentally, emotionally—I want to be the husband. I want to be the dad. I want to be the carer. The one who takes care of everyone else…I don't want to be a lesser person. (Male, white British, 50s)

Some participants expressed frustration at being ‘fussed over’ or ‘treated as an invalid’.

Then we got home and it was just, people were making a fuss of me which is nice but it gets a bit irritating because you're not, you know, you're not an invalid basically…people would make me cups of tea when they never made me cups of tea before and I kept assuring them that I was alright. (Male_2_, white British, 50s)

To preserve their identity and role after the illness, some participants were particularly keen not to disclose their illness to work colleagues.

To be honest I kind of made very little of it at work. I simply said, at each point of activity, ‘I'm going to be away for whatever. I need this, this and this done.’ And I think because of my prior cosmetic surgery the assumption was, oh he's off for another face lift. And I just let people believe whatever they wanted to believe. (Male_6_, white British, 50s)

In the retired group, self-esteem appeared to be more related to the physical limitations they had in activities of daily living.

Seven out of 10 Indian men gave an account which appeared to normalise their situation after their cardiac event, with reduced reporting of negative emotions.

I haven't got no problems. I'm as normal. I've never had any problem with anything before it was implanted, and after that, a completely normal life. I feel that nothing happened to me. (Male_8_, Indian, 60s)

### Perceived physical impact of CHD

Somatic symptoms across all three ethnic groups were common, particularly fatigue after the event—both in the short and long terms. There was reduced exercise tolerance for some with respect to walking or gardening, and a reduced ability to undertake domestic tasks. Some reported curtailing hobbies that involved physical exertion for fear of provoking a further cardiac event. This was particularly the case in the white British and Indian groups. The need for these changes had an impact on people's self-confidence.

I realised how tired I was and I didn't want to do anything else and I let people arrange things and do things, whereas I've never been like that, I've always, oh I can do it, you know. But I just let them all get on with it and I just felt very, very tired. (Female_4_, white British, 80s)

Many who were healthy before began to see themselves in a sick role in the long term, not just in the immediate aftermath of the acute cardiac event. There was a fear of heavy lifting across all three groups, irrespective of ethnicity and socioeconomic class, in the postdischarge period and in the year following the event. Participants and their families reportedly perceived the participant to be physically weaker after the cardiac event. Participants made practical adaptations (reducing housework, changing job roles) to their activities in daily life in order to adapt to their perceived or actual physical limitations.

When it comes to lifting stuff then I'm not allowed to do that.…I had got a little out of breath cutting the lawn and doing some vacuuming in the house, so I just have to take it easy. I rush around doing stuff. Maybe I just have to take it a little more leisurely. (Male_9_, white British, 80s)

This fear of heavy lifting is particularly relevant for the Bangladeshi group, which had a higher number of younger males with young dependent families, in manual jobs. There was little opportunity for alternative roles, impacting on their identity as the main breadwinner for their family.

Mentally I think I've had a heart attack. My doctor said I've got a weak heart, ‘Don't do this, don't do that…Obviously as a man, obviously if you have family, obviously the first thing you think about is money…So that's why I'm worried. So money worries financial worries sometimes. If you are very badly problem in financial then it makes you upset and you can put yourself in depression…I don't want to be dependent. I don't want for someone to support me. I feel humiliated…Like I'm just sitting down, sleeping, working, smoking, eating, and she's earning money and she's buying food for me. I don't want to do that. (Male_5_, Bangladeshi, 40s)

### Attitudes to future

Across the participants, among the working and non-working population in the Indian and white British group, there was a heterogeneity in personal attitudes to the event and how they adapted, with many reporting feeling lucky to be alive and optimistic.

If you, you know, you could sit around and grumble but you're only grumbling at yourself aren't you?…Don't think, don't dwell on your heart attack, get on with, your life I mean…(Female_4_, white British, 80s)

A positive attitude to the future was reported less by Bangladeshi participants, the majority of whom were of working age and from a more socioeconomically deprived background. Many in this group expressed financial concerns related to limitations in future employment options, which appeared to negatively impact on their overall views about the future. Only one working participant in the Bangladeshi group expressed optimism. This participant, however, differed from the other Bangladeshi participants as they had a skilled non-manual job and had attended higher education.

A number of participants across the three groups expressed a strongly fatalistic attitude to the future, irrespective of age, gender, working status, religion or socioeconomic group.

So always believe what happens by the Allah, praying, all these things, they are doing their best but everything in his hands, what I believe…Why should worry? If I need to go again. Worry make you worst don't it? If you worry about something, I don't take in the worry. But now what will happen will happen, no one can stop it. Why should I worry? (Male_10_, Bangladeshi, 70s)

Faith was expressed as being important for adaptation by many participants, primarily among the Bangladeshi group.

But I suppose it all depends how much faith you have on the Almighty. Since I strongly believe in the Lord, I have a strength within me to come over it, so people who, they have weak faith they're more worried, I suppose they are more worried than me…If you have strong faith that gives you strength in order to endure the situation and overcome it and adjust to it. (Male_12_, Bangladeshi, 60s)

### Social and professional support for adaptation

#### Family/friends

Most participants described getting excellent support from friends, family and work colleagues. Participants reported this led to increased morale, as well as provided help with physical and domestic tasks.

My son lives not far from my house. I see him every day. We're a close family. I've got brothers, sisters-in-law, nephews. We are very close. Just a phone call and they'll all be there. So that way I'm really happy. Lucky anyway…When you can count on somebody, even in the back of your mind, you relax. At least you know somebody cares and they are there when you need them. That makes all the difference to me. (Male_13_, Indian, 60s)

The difference a supportive, meaningful social network made for psychological adaptation after illness was illustrated by two female Bangladeshi participants, both widowed, living within an extended family in the same socially deprived area. One participant reported moderate low mood and the other reported no problems with mood.

With the first participant it appeared she felt help was given grudgingly by family members and she felt very much alone despite living in a large household.

She want liaison office…She's saying she can tell her house problem, if anyone abuse her, you know, tell her off and this that, to ask her to do that sort of thing. She's saying, no they're not doing it at the moment, she says, how long they keep doing, you know, looking after her, giving her food. She says one day they will get angry and might say something to her so she need to tell someone that sort of thing. That's why she need someone to talk to, someone like she can easily access. (Female_15_, Bangladeshi, aged 60s, moderate low mood, translated)

The second participant described an extremely supportive network.

‘Her family are taking care of her so she can, you know…She doesn't have to think about anything in the house, you know, financially, whatever, cleaning, cooking, doesn't have to worry about anything…They arrange everything…(Female_14_, Bangladeshi, aged 60s, no low mood reported, translated)

#### Professional service support

##### National Health Service: GP/hospital

In all three ethnic groups, very few saw GP services as a valuable source of psychological or practical support after discharge. Two participants in the Bangladeshi group reported rude, discriminatory or uncaring staff in particular hospitals. Some Bangladeshi individuals highlighted concerns that family were being used as interpreters, or reported ineffective interpreters who were formally employed by the National Health Service (NHS).

The white British and Indian groups, working in managerial or professional roles, were the predominant groups who reported using the internet, reading the leaflets provided or asking for advice from friends and family who were in the medical profession. Few Bangladeshis were able to read the leaflets or access the internet for more information.

I have a friend who was a cardiology nurse, so I had a chat with her. Also I looked it up on the internet. And I also spent some time reading the angiogram notes to see what condition I was really in. (Male_1_, white British, 50s)

##### Cardiac rehabilitation programme

Just under half of participants attended a cardiac rehabilitation programme, and the remainder reported actively declining or that they were not offered or were unaware of a cardiac rehabilitation programme. More participants from the white British group reported attending the programme, and more in the Bangladeshi and Indian groups stated they were unaware of the programme.

Of those who attended the cardiac rehabilitation programme, all but two participants found it to be a positive experience offering useful, indepth information on their condition. They reported it increased their confidence and that meeting peers and group therapy benefited them psychologically.

I see the difference in myself when I came out of the class. It was very useful…and to hearing the others…But the class actually helped me to understand more, and listening to other people's stories…Actually the education at that class actually is the main thing really helpful in every way, for the medication, or the exercise and the relaxation. That gave me more confidence and to relieve some of my fear. GPs don't have the time and everything. So this is actually three hours or two hours, 10 till 1, three hours. Really relaxed and get more information…(Male_9_, Indian, 50s)

Two of the elderly retired participants, one female Bangladeshi and one male white British participant, went on to have further heart attacks following exercise rehabilitation, and they attributed it to ‘overdoing it’ as a result of these sessions.

She says there's a place in XXX, I don't know, leisure centre or, she went there for two weeks and after that she had a heart attack, again. After the first episode she had a second one again, so that's why she didn't go. (Female_15_, Bangladeshi, 60s via translator)

These participants also expressed a fear of heavy lifting.

Of the eight participants who stated they were unaware of a rehabilitation programme, seven were from the more socioeconomically deprived Bangladeshi group. It is not known whether they were not offered the service, if there was no appropriate service for their condition, or if there was an issue with the language in which the service was provided.

### Practical adaptations made after the event

#### Return to work

Returning to employment was seen to present particular challenges. In the Indian and white British groups, especially among those in professional or managerial posts, the cardiac event prompted change in the nature of employment, for example, more part-time work or giving up a previous work role. They generally reported feeling supported in their work environment and were able to return to work in an adapted role.

Quite a lot of the work I was doing part-time means humping heavy stuff around, and I think a combination of being told maybe you're not as fit as you should be, and you can't lift anything, and also my back pain, so I've not gone back to doing that job. I've been given another job which is less strenuous, but even that, I don't work that often. It's very part-time, if you like. Mostly administrative. (Male_18_, white British, 60s)

There was flexibility in their roles and opportunity for social support from team members.

… And they was kind. They were saying, go home a bit early, don't overdo it, you know edge back into work and everything else. So it worked out very, very well … Colleagues at work were supportive. (Male_2_, white British, 50s)

This is in contrast to the more manual and solitary (eg, mini-cab driver) posts of the Bangladeshi workers. Only one of the five Bangladeshi participants who were working prior to the event was able to return to the original work role by 12 months postevent. The Bangladeshi group was predominantly from a younger age group with dependent families (only one retired participant). As discussed earlier, this group expressed more negative emotions and fear of the future as their perceived health status was seen as a barrier to returning to work. There were also fewer opportunities for alternative work or workplace adaptations that took into account their new circumstances.

Some of the Bangladeshi participants related their negative emotions to the fact they were at home all day with no purposeful employment. These participants were more likely to express feeling both physically and mentally weak.

Because every hours, every day, about five/six hours you're working you different life, you know, different thing, you're working. Always you sit in home, that's…doesn't help you feel particularly good? No, No. (Male_19_, Bangladeshi, 50s, long term unemployed)

#### Lifestyle changes

All three groups, independent of age, sex and socioeconomic background, focused on the importance of making active lifestyle changes and reported adopting dietary changes, with the Bangladeshi group particularly reducing red meat consumption. In some, concern about triggering a future heart attack prevented them from doing exercise at the same level as done previously.

I did become very much careful about the red meat, avoiding the fatty food and all that…I walk a little bit, I go for walk, although I can't walk much…being careful what I eat, you know…since then I have taken some effort myself of taking some medicines. (Male_16_, Bangladeshi, 61 years)

Participants from all three ethnic groups and from all socioeconomic backgrounds reported that they would like access to psychological support (group therapy or individual one-to-one therapy) to aid adjustment and improve mental health following their cardiac event.

If there are any people that you can consult or talk to, I'm a) not aware of anybody, and b) I think they have to be kind of informally accessible. I think it's a barrier if you have a lot of form filling and red tape and stuff to go through. If you're able to say, ‘Look, can I come and have a chat…It would be enormously helpful if there was a local group, a local person that understood the conditions and the treatments. (Male_6_, white British, 50s)

## Discussion

Across the three groups, participants showed heterogeneity in reported experiences of the cardiac event, and its subsequent psychological and physical impact. Adaptation to the event, both psychologically and physically, did not appear to relate to the severity of the initial event, but varied by socioeconomic status, age and ethnicity.

Low mood, anxiety, fatigue and fear of heavy lifting following the cardiac event were themes common to all. There were differences in the psychological adaptation experienced after the cardiac event between the ethnic groups, which was partly explained by differences in socioeconomic status and occupations prior to the cardiac event.

Participants in the Bangladeshi group (with the younger mean age of 58 years, and from more manual occupations) tended to experience more mental health issues alongside physical symptoms, especially fatigue. This was strongly associated with difficulties with returning to employment and associated financial concerns, with resultant negative impact on self-esteem. Some members of this group were fatalistic; in particular, they made reference to their faith, saying, for example, ‘Allah will look after me’. This belief appeared to help acceptance and adaption to their new circumstances after the cardiac event. In the male Indian group, most reported less low mood and fewer physical health symptoms in comparison to the white British and Bangladeshi groups, with a tendency to normalise the event in their accounts.

### Comparison with existing literature

There is little qualitative literature exploring the experiences and impact of heart disease for UK South Asian populations. Research has shown that job uncertainty is linked to deterioration in health status.^21^ This is likely to be a factor in the negative adaptation following a cardiac event that we found in men in the Bangladeshi group.

Older Indian males in our study appeared to show more positive adaptation, and attempted to normalise the experience. Evidence from previous studies show they have worse outcomes in terms of secondary cardiac events after angina management than the white British.^22^ It is possible that there is more positive adaptation psychologically, but there is an increased genetic risk^2^ ^4^ or that the normalisation means they make fewer efforts to change unhealthy lifestyle behaviours. The latter is supported by a qualitative study based in Leicester which examined the experiences and needs of one ethnic group—Hindu Gujarati patients and partners postmyocardial infarction, which highlighted a lack of lifestyle changes.^23^ Similar findings of non-adherence to diet or other lifestyle changes by UK South Asians were seen in two other qualitative studies^24^ ^25^ which had examined South Asian attitudes to lifestyle coronary risk factors and reported that lifestyle changes were not made or not adhered to.

In our study, fatigue, as a multidimensional concept involving tiredness, weakness and lack of energy, was a common theme. This may be a somatic expression of the negative psychological effect of the event, or physical sequelae related to reduced heart functioning (heart failure) due to the event. There is evidence to support this as a widespread issue in patients with long-term illnesses, including CHD.^26^ Our study found that the fear of heavy lifting, which persisted, limited full physical rehabilitation long term. This aspect postcardiac event does not appear to be raised in similar qualitative studies.^27^ Fear of lifting may be due to conflicting or ambiguous medical advice.^28^

The need for individually tailored rehabilitation is supported by other studies;^24^ ^25^ this highlighted a need for a tailored individual programme for South Asians which avoids stereotyping, but recognises cultural barriers to change. However, the former study also cautioned that not every difficulty can be attributed solely to the person's ethnic background and similarly, we found that apparent initial ethnic differences in psychological adaptation in our Bangladeshi group were explained to a large extent by the much greater problems of returning to work that is related to the types of employment and the younger age of this group. Our study is in keeping with others^23^ ^29^ that have reported socioeconomic status, age and gender are as important as ethnicity when examining barriers, psychological or physical, in adapting to cardiac disease. Indeed, it has been argued that for long-lasting change, cardiac prevention initiatives need to incorporate cultural sensitivity while also taking into account the socioeconomic circumstances of ‘at-risk’ communities.^30^ ^31^ This appears especially pertinent to our Bangladeshi group. A lower number of Bangladeshis compared with other groups in our sample reported attending the cardiac rehabilitation programme, and most who had not attended were unaware of the existence of these services. Given that most of the participants who had attended cardiac rehabilitation reported that it was beneficial, this lack of awareness may also be a factor in the more problematic psychological impact and adaptation after the cardiac event in the Bangladeshi group.

### Strengths and limitations of the study

We purposively sampled a range of participants from white British, Indian and Bangladeshi participants, and explored experiences and attitudes of the psychological impact and adaptation after a cardiac event. We sampled to ensure a broad range of views and experiences from patients within a wide range of ages, working and retired, and from a variety of socioeconomic backgrounds and education. Our Bangladeshi participants were on average younger than our white participants (mean age 58 vs 72 years), and factors associated with their age, such as employment status and dependent family members, may have influenced their experiences and adaptations, and thus may account for the apparent differences between the groups. We acknowledge that the broad range of ages included may mean generational differences that may have had an impact on our findings. A strength of our study was that although we used trained interviewers who spoke Sylheti to make it easier for the participants to talk at length in their own language, the main researcher could also understand Bengali and the related dialect Sylheti. The researcher understood the interpreter interviewing the participants in Sylheti, allowing her to monitor the discussion during the interview, thus helping to ensure effective probes and follow-ups were used, and also to verify the accuracy of the interpreter's work. There is potential for sampling bias as the more motivated and proactive, less depressed participants may have agreed to take part. Moreover, women were under-represented; we were able to recruit two female Bangladeshi participants (a group known to be hard to recruit); however, there were no Indian women in the sample. Recruitment proved to be a particular challenge especially among females and Bangladeshi and Indian groups, which was possibly due to cultural or literacy issues as the initial approach was from their GP practice. The South Asian community is culturally heterogeneous; therefore, caution is needed with data interpretation on cultural issues. Within subgroups, there are differences in lifestyle, diet, alcohol consumption, religious and physical differences. The findings do, however, offer important insights for cardiac rehabilitation across ethnic groups.

### Implications for clinical practice and research

In this study, there was evidence of greater negative adaptation to a cardiac event in the Bangladeshi group with younger men especially perceiving difficulties returning to employment following the event. This was not seen to be adequately addressed in their rehabilitation, and more tailored help and support in relation to returning to work should be considered. Participants from all ethnic groups reported concerns about fatigue, and expressed anxiety regarding heavy lifting and physical exertion in the long term. Further research should evaluate if this is a consistent finding in a larger representative sample across different black and minority ethnic groups.

## Conclusions

Overall, successful adjustment after an acute cardiac event in our sample was related to socioeconomic background, age and individual personalities, with some cultural influences. Current rehabilitation services and follow-up in primary care should consider the differing psychological and physical changes, and adaptations that may be needed in the longer term, especially with regard to physical activity, heavy lifting and fatigue levels. Bangladeshi participants, in particular, experienced less adaptation which was related to difficulties in returning to employment and associated financial hardship. There is a need for extra support (psychological, practical and financial) with specific focus on working-age people with families returning to manual work.
